# Role of non-contrast spiral computerized tomography in acute ureteric colic

**DOI:** 10.4103/0970-1591.32059

**Published:** 2007

**Authors:** S Feroze, Baldev Singh, T. Gojwari, S. Manjeet, Bashir Athar, Hussain Hamid

**Affiliations:** Department of Radiodiagnosis, Sher-i-Kashmir Institute of Medical Sciences, Srinagar, India; * Department of Urology, Sher-i-Kashmir Institute of Medical Sciences, Srinagar, India

**Keywords:** KUB, noncontrast helical computerized tomography, ureteric calculi, ultrasonography

## Abstract

**Aim:**

To evaluate the sensitivity and specificity of noncontrast helical computerized tomography (CT) in ureteric colic with comparative evaluation of KUB and ultrasonography (USG).

**Setting:**

Tertiary care university hospital.

**Materials and Methods:**

One hundred patients aged between 20 and 75 years referred from the emergency department as acute ureteric colic were evaluated with KUB and USG followed by noncontrast helical CT.

**Results:**

Noncontrast helical CT was 91% sensitive and 98% specific in detecting urolithiasis compared to a sensitivity of 20% and 30% for KUB and USG and specificity of 94% and 98% respectively.

**Conclusion:**

Noncontrast helical CT is a very sensitive and specific investigation for evaluation of acute flank pain due to urolithiasis, besides helping in the detection of nonrenal causes of pain.

Acute ureteric colic is one of the most common emergency admissions and needs an investigation which is sensitive, specific and quick to perform, not only to confirm urolithiasis but also to exclude serious nonrenal conditions in need of immediate intervention. Noncontrast helical computerized tomography (NCCT) fulfils most of these requirements.

## MATERIALS AND METHODS

One hundred patients aged 20-75 years were referred from the Emergency Department over the last two years for evaluation of acute urteric colic. Out of 100 patients 68 were male and 32 were female with median age of 38 years in males and 33 years in females.

These patients were referred from casuality and had ureteric colic ranging in duration from a few hours to a maximum of 36h presenting first time or as second or third episode of ureteric colic. All the patients had plain KUB and ultrasonography (USG) followed by NCCT. The machine used for KUB was 500 mAs GE Wipro (nondigital), NCCT of whole abdomen was done on Siemens Emotion spiral CT with 8 mm slice thickness and 4 mm recon increment. Findings were evaluated by radiologist. Patients with positive and/or equivocal findings on KUB and USG (e.g., dilated pelvicalceal system) or negative results from the above modalties were subjected to NCCT. The radiologist knew the findings of these tests beforehand and he confirmed or negated the findings on NCCT. Besides direct signs of urolithiasis, indirect signs like hydronephroris, hydroureter and peri-ureteric or peri-nephric stranding were also recorded.[14] The findings were confirmed on operative retrieval or spontaneous passage. Patients after emergency NCCT were followed up in OPD for spontaneous passage, persistence or aggravation of symptoms. All these cases were followed for a few months to 18 months depending upon whether the stone was passed spontaneously or the patient was subjected to surgical intervention.

## RESULTS

**Figure 1:**
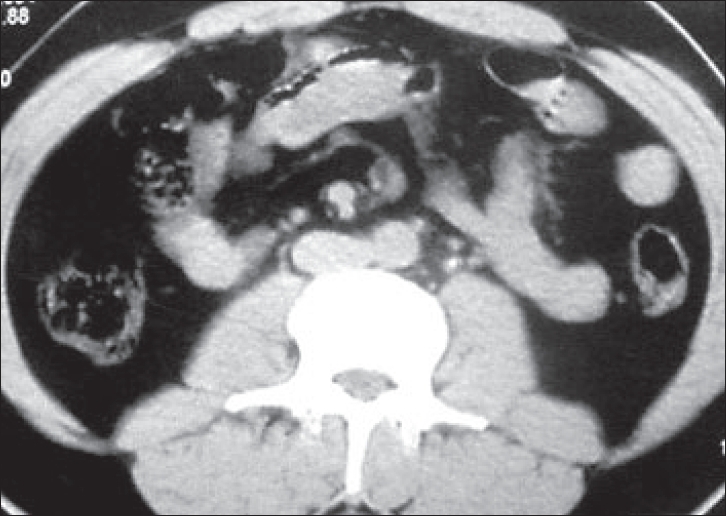
Non-contrast computed tomography showing left ureteric calculus

**Figure 2:**
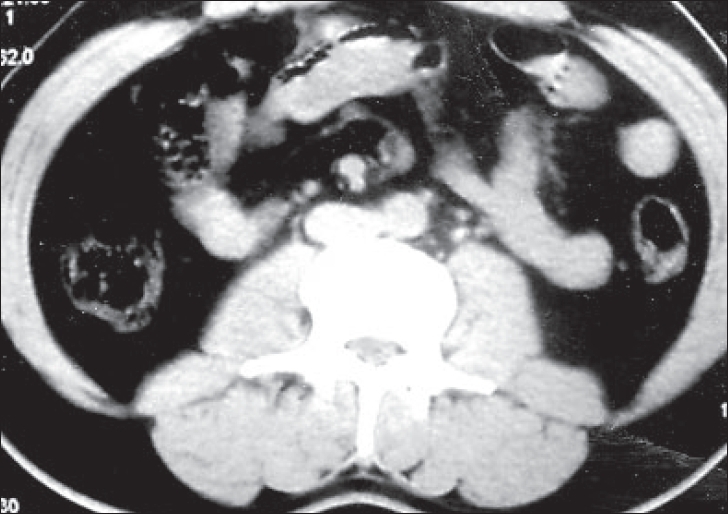
Non-contrast computed tomography showing right ureteric calculus

**Figure 3:**
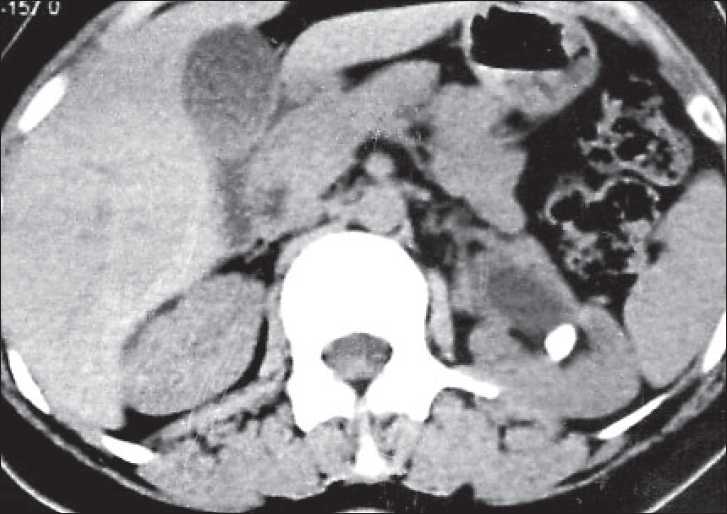
Non-contrast computed tomography showing left renal pelvic calculus

Urolithiasis was found in 20 patients on plain KUB, of which 14 constituted ureteric calculi. The USG showed direct evidence of urolithiasis in 27, renal calculi in 14, ureteric calculi in nine and both in four, the size of stone varied from 3 mm to 24 mm with median size 11 mm. Most calculi detected on USG were either at the pelviureteric or at the vesico-ureteric junction. Indirect signs of urolithiasis were seen in 36%. NCCT detected calculi in 40 patients, both ureteric [Figures 1 and 2] and renal [Figure 3] in 17 and only ureteric calculi in the rest of the 23 patients.

In our study the true incidence of ureteric calculi was 43 on the basis of spontaneous passage or ureteroscopic removal. Twenty patients out of a total of 40 who had calculi on CT were missed on X-ray KUB and had median size of 9 mm, both ureteric as well as renal. The NCCT showed false positive result for stone in one patient, the ultrasound showed for one patient and KUB for three patients who had no stones.

Out of 40 patients who were labeled as having renal/ureteric calculus on NCCT one patient had renal parenchymal calcification and USG defined it better and easily whereas it was indistinguishable on NCCT and was taken as false positive. Variation in result due to different age group did not affect our study by NCCT, however, USG findings were difficult to obtain in obese patients.

Comparative sensitivity of KUB, USG and NCCT was 20%, 30% and 91% and specificity 94%, 98% and 98% in that order.

## DISCUSSION

The conventional modalities of ureteric colic/ flank pain investigation are plain KUB, ultrasound and IVU. In one study plain KUB had sensitivity and specificity of 45% and 77% respectively.[1] USG alone has a sensitivity of 56%.[2] USG alone missed stones < 5 mm in diameter, the majority of them located in the middle and lower ureter. IVP was 91% sensitive.[2] Combined use of USG and KUB compared to IVP revealed sensitivity of 95% but was less specific i.e., 67%, suggesting that IVP rarely provides additional information when the combination of KUB and USG is negative for detecting calculi.[3]

In our study KUB had a sensitivity and specificity of 20% and 94%, USG had sensitivity and specificity of 30% and 98% respectively. USG was valuable in detecting extra-renal pathology in 7% of patients. USG revealed renal stones in 14 out of total 27 positive cases and nine ureteral stones and both renal and ureteral stones in four patients. Most of the stones seen on USG were at upper ureteral region (54%) and at UV junction (23%). Secondary signs of ureteral stones were seen in 36 patients and were hydronephrosis (69%), hydroureter (61%) and both (15%). Extrarenal pathologies seen were acute appendicitis (1), acute cholecystitis (3), CBD calculi (1) and adrenal mass (1).The patients enrolled in our study were those with signs and symptoms of classical ureteric colic thus attributing for lesser sensitivity of USG (30%) compared to other studies which included patients with nonspecific flank pain. Thus all patients with renal calculi and s/s of ureteric colic had ureteric calculi as proved on operative retrieval or spontaneous passage.

Among all the 100 patients, NCCT showed urolithiasis in 40 patients and extra-urinary pathology in 12 patients. Forty-eight patients were normal. Of 40 patients, with calculi there were 40 ureteric and 17 combined renal and ureteric. Secondary signs of urolithiasis were seen in 38 patients i.e., hydronephrosis in 26, hydroureter 26. Twenty-five patients had perinephric stranding and rim sign. Out of 100 patients in our study true incidence of ureteral stones was 43 as determined by operative retrieval in 40 patients and spontaneous passage in three patients. Fifty-seven patients did not demonstrate any stones out of which 12 had pathologies unrelated to urolithiasis. In the remaining 45 patients, cause of flank pain could not be ascertained. Thus statistical analysis shows NCCT had sensitivity, specificity, PPV, NPV of 91%, 98%, 97% and 93% [Table 1].

**Table 1:** The comparison with other series reports is given as

Name of the study	Sensitivity	Specificity	PPV	NPV
Smith *et al*, 1996[4]	97	96	-	-
Fielding *et al*, 1997[5]	98	100	-	-
Miller *et al*, 1998[6]	95	98	-	-
Dalrymyple *et al*, 1998[7]	95	98	-	-
Yilmaz *et al*, 1998[8]	94	97	98	98
Chain *et al*, 1999[9]	100	94	93	-

NPV -Negative predictive value, PPV - Positive predictive value, Figures in parentheses are in percentage

Shreyer 2002,[10] similarly Marineck 2002[11] and Tamm *et al* 2003[12] in their comments describe the high accuracy rate of helical CT scan in detecting urolithiasis even at low doses.[13] In conclusion helical noncontrast CT is a sensitive, specific and quick investigation for evaluation of urolithiasis, with additional benefit of detecting nonurinary causes of flank pain. Latest protocols of low-dose CT would further enhance its utility.

## CONCLUSION

Plain KUB and USG are less sensitive than NCCT although specificity is almost the same. USG diagnosed 27 cases and missed 13 cases whereas NCCT diagnosed all 40 cases. We recommend NCCT in all cases of clinical findings of urerteric colic where plain KUB and USG are negative or equivocal.
